# Impact of opinion dynamics on recurrent pandemic waves: balancing risk aversion and peer pressure

**DOI:** 10.1098/rsfs.2024.0038

**Published:** 2025-09-26

**Authors:** Sheryl L. Chang, Quang Dang Nguyen, Carl Joseph Edmund Suster, Ma Christina Jamerlan, Rebecca J. Rockett, Vitali Sintchenko, Tania C. Sorrell, Alexandra Martiniuk, Mikhail Prokopenko

**Affiliations:** ^1^ Centre for Complex Systems, The University of Sydney, Sydney, New South Wales, Australia; ^2^ Sydney Infectious Diseases Institute, The University of Sydney, Sydney, New South Wales, Australia; ^3^ Westmead Hospital Centre for Infectious Diseases and Microbiology - Public Health, Wentworthville, New South Wales, Australia; ^4^ Faculty of Medicine and Health, The University of Sydney, Sydney, New South Wales, Australia

**Keywords:** opinion dynamics, recurrent waves, pandemic modelling, COVID-19, social distancing, infectious disease

## Abstract

Recurrent waves, which are often observed during long pandemics, typically form as a result of several interrelated dynamics, including public health interventions, population mobility and behaviour, varying disease transmissibility due to pathogen mutations, and changes in host immunity due to recency of vaccination or previous infections. Complex nonlinear dependencies among these dynamics, including feedback between disease incidence and the opinion-driven adoption of social distancing (SD) behaviour, remain poorly understood, particularly in scenarios involving heterogeneous population, partial and waning immunity and rapidly changing public opinions. This study addressed this challenge by proposing an opinion dynamics model that accounts for changes in SD behaviour (i.e. whether to adopt SD) by modelling both individual risk perception and peer pressure. The opinion dynamics model was integrated and validated within a large-scale agent-based COVID-19 pandemic simulation that modelled the spread of the Omicron variant of SARS-CoV-2 between December 2021 and June 2022 in Australia. Our study revealed that while holding epidemiological factors constant, the fluctuating adoption of SD, shaped by individual risk aversion and social peer pressure from both household and workplace environments, can reproduce these multi-wave patterns, pointing to the importance of social dynamics in understanding epidemic outcomes.

## 1. Introduction

The presence of recurrent waves of infections [1,2] has been reported in pandemics of several pathogens [3–5]. For influenza, multiple waves have been noted as a distinguishing feature of pandemics, and an opportunity for informing public health responses [6]. Accordingly, there is much interest in understanding contributing factors that can explain the observed pattern of waves [7–10]. Seasonal effects, evolutionary dynamics of the pathogen, and interactions with other co-circulating pathogens are each important; however, many of these explanatory models focus on the impact of human behaviour. The contact patterns of infected hosts with immunologically naive populations often determine early pandemic dynamics.

As a pandemic progresses, populations observe the impacts of both the disease and the implemented control measures. Over time, control measures differ in the extent to which they impose on individual freedoms, ranging from mandated interventions (e.g. lockdowns, border closures, quarantine) to less prescriptive approaches (e.g. recommendations and education campaigns). Although the framework of an ‘ethical intervention’ may vary across different people and nations, voluntary non-pharmaceutical interventions will inherently have trade-offs between individual freedoms and potential benefits. Arguably, ethical interventions should account for impacts on an individual’s *freedom* to move and interact with others without unwarranted external restrictions, and *fairness* in the distribution of benefits and restrictions [11]. When mandatory restrictions are progressively lifted, individual adherence to the interventions shifts from *compliance* to voluntary *adoption*. This change allows individuals to adapt their decisions and adjust their interactions within the population (e.g. social distancing (SD)) based on their own risk assessment rather than government restrictions. Public health ethics present new challenges to infectious disease modelling, prompting modellers to take into account individual risk assessment and the dynamics of opinion formation [12]. For example, changes in risk assessment and the resulting behavioural shifts are closely linked to the emergence of recurrent epidemic waves [13].

A pattern of recurrent waves of infections was observed during the COVID-19 pandemic in many countries around the world by many variants [2,14,15]; in Australia, between December 2021 and June 2022, the Omicron variant was dominant. During this period, the population had a relatively high vaccine coverage (i.e. over 90% within the adult population) [16–18]. This Omicron wave of infections resulted in a prominent peak reaching 105 000 new cases per day around early January 2022 (over 4100 cases per million), followed by two subsequent smaller peaks reaching 66 000 new cases per day around early April 2022 (over 2500 cases per million) and 59 000 new cases per day around late May 2022 (approx. 2300 cases per million), followed by two subsequent smaller peaks reaching 66 000 new cases per day around early April 2022 (over 2500 cases per million) and 59 000 new cases per day around late May 2022 (approx. 2300 cases per million) [19]. The persistence of the Omicron variant during this period has been partially attributed to the fluctuating adoption of non-pharmaceutical interventions (NPIs), particularly SD, with individuals adjusting their behaviours in response to the pandemic severity [20].

Using our census-calibrated agent-based model (ABM), we previously identified a plausible sequence of step changes in the fraction of the population adopting SD (i.e. SD profile) which reproduced the observed pattern of recurrent waves over a period of approximately 29 weeks when Omicron variant was dominant [20]. The SD profile was determined retrospectively by tuning the step changes to minimize the difference between simulated and actual disease incidence. The resultant profile included an initial period of low SD adoption fraction (30% of the population), followed by a period of relatively high SD adoption (70% of the population) when the incidence approached the first peak, a rapid decline to a moderate level of SD adoption (by 50−60% of the population) during the second wave and a drop to low SD adoption (at 30%) around the third mid-year wave. We hypothesized that declining SD adoption could be attributed to pandemic fatigue [21]. Although our prior study [20] revealed an important relationship between SD adoption and pandemic spread, it did not explicitly model risk assessment and the dynamics of opinion formation that lead to voluntary SD adoption, nor did it examine the key factors that influence changes in social behaviour.

In this study, we aimed to develop a model of opinion formation and dynamics which can shape complex, voluntary SD adoption behaviours without relying on *post hoc* fitting to observation. Previous studies have modelled opinion dynamics using two perspectives: (i) opinion formation based on personal beliefs (e.g., individual risk evaluation due to fear of infection [22–24], or perception fatigue [25]); and (ii) social influence (e.g. imitation [26,27], averaging opinions [28] or majority-following opinions [29]). Our opinion formation model incorporates both of these aspects into our pandemic ABM. At each simulated cycle, the current opinion of each agent depends on their perceived level of risk (risk aversion [30]) and on the opinions of the agents they interact with (peer pressure). An agent’s opinion determines its behaviours on SD adoption. We quantified the impact balancing risk aversion and peer pressure on opinion dynamics, and explored how a combination of the individual and social perspectives can reproduce the recurrent pandemic waves. In doing so, we identified the effect on opinion formation of varying memory horizon, perception fatigue, and social contexts.

The remainder of the paper is organized as follows. We describe the ABM, including the pandemic transmission and control, i.e. NPIs and vaccination rollout (§2), and the opinion dynamics model (§2.1). We then report the key factors contributing to opinion dynamics that generate recurrent pandemic waves (§3). Finally, we discuss the limitations and implications of our study (§4)).

## 2. Methods

We simulate the viral transmission of the Omicron variant of COVID-19 in Australia, as well as pandemic control measures (NPIs and vaccination roll-out), using a high-resolution AGM which was previously calibrated and validated [20,31–34]. Our model comprises 25.4 million anonymous agents, each assigned several demographic attributes derived from the latest 2021 Australian Census [35], so that the artificial population represents the key demographic characteristics of Australia. These demographic attributes, including age, gender, residential area, student enrolment and workforce/educational groups, also determine the social contexts in which agent-to-agent interactions and thus, disease transmission, take place [34,36].

In our model, disease transmission follows a discrete-time simulation, updating each agent’s health state over time: susceptible, latent, infectious (asymptomatic or symptomatic) and recovered. The simulation begins with infections seeded around international airports (mostly in metropolitan areas) and continues to spread within the artificial population through interactions within multiple social contexts. These interactions are simulated in two phases per workday with different contact and transmission rates: ‘daytime’ cycle where interactions take place in workplace or education contexts (e.g. class, grade, school), and ‘night-time’ cycle where interactions take place in residential context (e.g. household, household cluster, neighbourhood and community). On weekends, interactions are assumed to occur solely within residential contexts, consisting of two ‘night-time’ cycles instead [34].

The probability of an exposed agent becoming infected is adjusted by checking if the agent is (i) vaccinated prior to the start of the pandemic wave, and/or (ii) adopting or complying with NPIs, such as SD, case isolation and so on. Out of all infections, only a fraction is assumed to be detected, to match the voluntary self-reporting system adopted in Australia during the Omicron stage. Here, we approximate the case detection rate based on the prevalence of anti-nucleocapsid antibodies in Australia [37] (see appendix A for more details).

Following previous studies [20,32–34], we assumed a high level of pre-emptive vaccination coverage in the population (90%) with two types of vaccines (priority vaccine, with a higher efficacy; and general vaccine, with a lower efficacy) distributed prior to the start of the simulation (i.e. the start of the Omicron stage in December 2021), in line with the reported vaccination coverage and vaccine distribution in Australia [16]. In addition, we introduced a waning immunity (from both vaccination and previous infections) into the model by tracking the vaccination and infection record of each agent, with the immunity declining over time (see appendix A). Consequently, in this extension of our model, agents can be re-infected after recovering.

NPI adoption reduces disease transmission by reducing the strength of interactions between agents across various social contexts. These NPIs include: case isolation (CI, affecting symptomatic infectious agents and detected asymptomatic agents), home quarantine (HQ, affecting household members of infected agents), SD (affecting susceptible agents) and school closures (SC, affecting school-aged agents, their households and teachers). Given our focus on the interplay between opinion dynamics and recurrent pandemic waves, we model SD adoption as a voluntary, opinion-driven decision which may change as the pandemic progresses (while assuming static adoption with other NPIs). Here, SD is modelled as a generic intervention which reduces the overall intensity of agent interactions, intended to capture the combined effect of reduced travel, mask-wearing, and physical distancing (see §2.1).

### 2.1. Opinion dynamics

**Figure 1.**
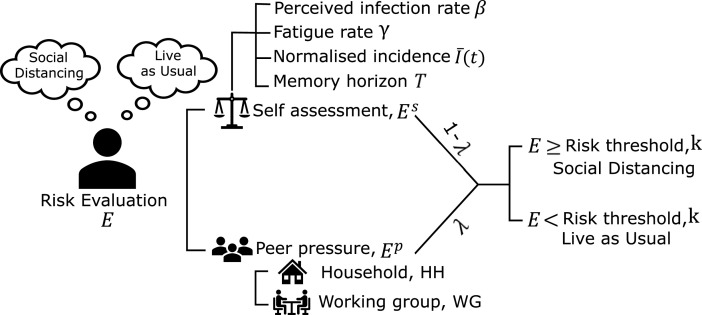
Opinion dynamics model overview.

We divide the population into three groups of agents: (i) committed, compliant agents which always comply with SD (25% of the population, assumed and randomly assigned); (ii) non-compliant agents which never comply with SD, due to being either an essential worker or a contrarian (25% of the population,^1^ randomly assigned); and (iii) rational agents which change their SD adoption based on the current pandemic severity (the remaining 50% of the population). A rational susceptible agent has a choice between two behaviours: (i) ‘live as usual’ behaviour, and (ii) ‘socially distancing’ behaviour. A rational agent may choose to switch between these two behaviours at any time as a result of the risk evaluation process following three steps: (i) self-evaluation where the opinion on SD adoption is formed individually by assessing the risk of infection (see §2.1.1); (ii) partial peer pressure where each agent may be partially influenced by opinions of the agents in their social contexts (see §2.1.2); and (iii) integration of the self-evaluation and peer pressure steps using a weighted average (see figure 1).

#### 2.1.1. Self-evaluation

In self-evaluation, the susceptible rational agents form their opinions on SD following a simple risk evaluation. For a rational agent i, the perceived risk of infection, $E_{i}^{\text{s}}$, is compared against a risk threshold k. When the perceived risk of infection exceeds the risk threshold ($E_{i}^{\text{s}}\geq k$), agents adopt SD by reducing their interactions within the relevant social contexts (see appendix A). Conversely, when the perceived risk of infection is lower than the threshold ($E_{i}^{\text{s}}<k$), agents choose to live as usual and do not adjust their interactions. $E_{i}^{\text{s}}$ follows a dynamic process dependent on the incidence record on day d:


(2.1) $$E_{i}^{\text{s}}(d,T)=1-(1-\beta {)}^{\bar{I}(d,T)},$$

where β∈[0,1] is the perceived probability of infection that quantifies the agent’s attitude towards risk aversion. A higher β leads to higher $E^{\text{s}}$, so that agents become more likely to adopt SD. $\bar{I}(d,T)$ is the normalized moving average of recent daily incidence data:


(2.2) $$\bar{I}(d,T)=\frac{N^{\text{c}}}{N^{\text{n}}}\frac{1}{T}\sum_{\tau =0}^{T-1}I(d-1-\tau ).$$

$\bar{I}(d,T)$ averages the daily incidence, I(d), between day (d−T) and day (d−1). T acts as a memory horizon, limiting the effect of earlier incidence data on an agent’s behaviour. $N^{\text{c}}/N^{\text{n}}$ is the relative size of a typical community, defined by the ratio between the typical community size $N^{\text{c}}=10^{3}$ and the national population size $N^{\text{n}}=25.4\times 10^{6}$, according to 2021 census [35]. The choice of community size is informed by two major population resolutions used in the Australian census: SA1 and SA2, with their population means of 400 and 10 000, respectively. Since SA1 and SA2 are separated by two orders of population magnitude, we chose a typical community size between these two resolutions. Informally, this normalization converts the global incidence to the local level at which agents perceive the disease severity (i.e. at a population magnitude above the street level but below the local government area level).

**Figure 2.**
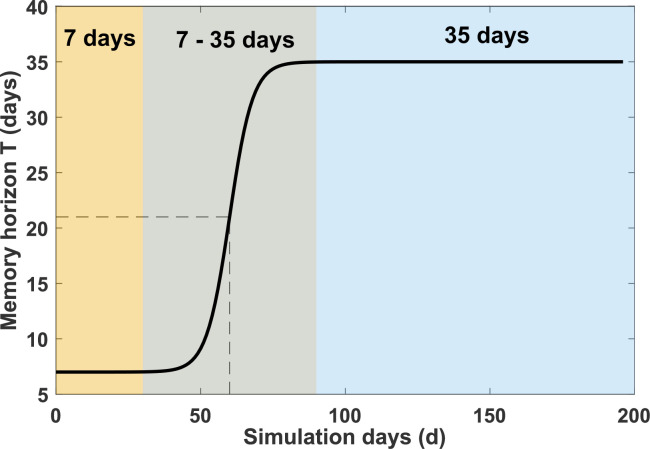
Sigmoid function for varying memory horizon, T(d), in equation (2.3). Shaded areas show the initial period with memory horizon of 7 days (yellow colour), the intermediate period of increasing value (grey), and the period with the final maximum value of 35 days (blue). Dashed lines mark the midpoint value of 21 days reached on simulation day d=60.

In the baseline model, T is a fixed parameter. We also consider an extended model where T is replaced by T(d), which varies globally throughout the pandemic simulation to account for the difference between time scales in transmission (i.e. faster scale) and behavioural change (i.e. slower scale). At the start of the pandemic, T(d) is shorter due to the lack of information on the past incidence, so that the agents use their immediate experience to evaluate the risk of infection. As the disease spreads, agents use a longer memory horizon for risk evaluation. The evolution of the memory horizon is modelled using a sigmoid function:


(2.3) $$T(d)=\frac{u}{1+{\text{e}}^{-v(d-L)}}+T(0),$$

where $T_{0}$ is the baseline memory horizon length at the beginning of the simulation, L, determines the simulation day when the memory horizon reaches its midpoint value, and u and v determine the shape of the transition region in the sigmoid function. We fixed these parameters to T(0)=7, L=60, u=28 and v=0.25, resulting in T(d) ranging from 7 to 35 days as illustrated in figure 2.

Finally, we consider the effect of pandemic fatigue, with the public attention to pandemic incidence declining over time [21,40], so that the individuals gradually become less alert to the current pandemic spread and consequently, less motivated to adopt SD. In the baseline model, the parameter β is fixed. We consider an extended model in which we reduce the risk aversion over time by replacing β with β(d) in equation (2.1), governed by the perception fatigue rate γ:


(2.4) $$\beta (d)=\beta_{0}(1-\gamma {)}^{d},$$

where $\beta_{0}$ is the initial value of the perceived probability of infection at the start of the simulation. The inset in figure 11b in appendix A shows how β(d) declines over time with γ=0.0002. Note that for γ=0, the time dependency is removed since $\beta (d)=\beta_{0}$, recovering the initial constant form. Henceforth we assume that β=β(d) and distinguish the baseline model by setting γ=0.

#### 2.1.2. Partial peer pressure

Individual opinion formation is known to be affected by opinions of other members of the community. Individuals tend to filter and integrate the information received, and align their opinions with those of their contacts in close social circles [41,42]. The peer pressure is modelled by accounting for the average opinion of an agent’s social context. We compute the perceived risk of infection of an agent i under peer pressure ($E_{i}^{\text{p}}$) as the weighted sum of the average opinions in relevant opinion-affecting social context:


(2.5) $$E_{i}^{\text{p}}(d,T)=\sum_{g\in G_{i}}\psi_{g}\frac{1}{|A_{g}|-1}\sum_{j\in A_{g}\setminus \{i\}}E_{j}^{\text{s}}(d,T),$$

where $E_{j}^{\text{s}}(d)$ as before is the perceived risk of infection of agent j who belongs to the same context g as agent i, $A_{g}\backslash \{i\}$ is the set of all agents in context g excluding agent i, and $\psi_{g}\in \left[0,1\right]$ is the relative weight of the context g in all social contexts $G_{i}$ that agent i belongs to, satisfying $\sum_{g\in G_{i}}\psi_{g}=1$. In our study, we considered two representative types of opinion-affecting social contexts: households (HH) and working groups (WG). Following [28], we model a combined perceived risk of infection quantified as a weighted sum of both self-evaluation ($E_{i}^{\text{s}}$, equation (2.1)) and peer pressure ($E_{i}^{\text{p}}$, equation (2.5)) components, in accordance with the Friedkin–Johnsen model [43]:


(2.6) $$E_{i}(d)=\lambda E_{i}^{\text{p}}(d)+(1-\lambda )E_{i}^{\text{s}}(d),$$

where λ∈[0,1] is the weight of peer pressure and 1−λ is the weight of self-evaluation in forming the current opinion.

For each rational agent i, the combined perceived risk of infection, $E_{i}$, is then compared against the risk threshold k, resulting in either adopting SD ($E_{i}\geq k$), or living as usual ($E_{i}<k$). For simplicity, we assume that all rational agents in the population share the same risk threshold. The SD adoption reduces interactions between agents in the same social contexts, thus reducing the infection probability $p_{i}(d)$ for SD-adopting agents i on day d. Appendix A.3 details how SD adoption affects the probability of infection.

## 3. Results

We present results in two parts, following the risk evaluation steps: (i) self-evaluation only (§3.1), and (ii) both self-evaluation and partial peer pressure (§3.2). To establish a baseline for evaluating the epidemic dynamics without opinion dynamics, we simulated (a) a null case with no agents adopting SD (i.e. SD adoption of 0), and (b) four control cases specified by fixing the SD adoption level at [0.25, 0.5, 0.75, 1.0], where SD adoption of 1.0 represents full adoption. Figure 10 in appendix A illustrates that all controlled and null cases failed to generate the recurrent waves observed during the Omicron stage.

We note that the simulated dynamics considered in this section are generated using 25 simulation runs. We demonstrated an adequate level of accuracy attained at this sample size, with the mean absolute percentage error being less than 5% when compared against the simulated incidence obtained from 100 runs (see figure 9 and appendix A.7 for more details).

### 3.1. Self-evaluation

We first evaluated the impact of opinion dynamics formed only by using self-evaluation. Specifically, we examined three scenarios: SE1, the baseline model; SE2, where only the memory horizon varies with time, i.e. T=T(d) and γ=0; and SE3, where both the memory horizon and the perceived probability of infection vary globally with time, i.e. T=T(d) and γ=0.002.

**Figure 3.**
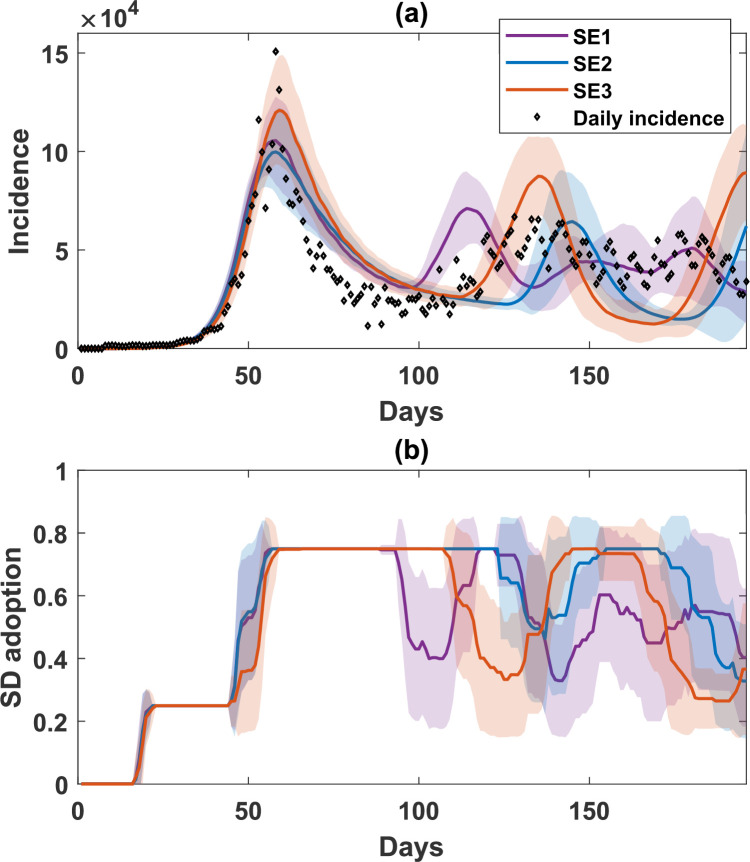
A comparison of simulated incidence (a) and SD-adoption fraction (b), with the opinion dynamics produced by self-evaluation only (SE1, baseline in purple; SE2, with changing memory horizon in blue; and SE3, with changing horizon and fatigue in orange). Refer to table 1 for parametrization. Shaded areas around the solid line show s.d. Each simulated profile is averaged over 25 runs. The actual daily incidence between December 2021 and June 2022 is shown in scattered diamonds.

**Figure 4.**
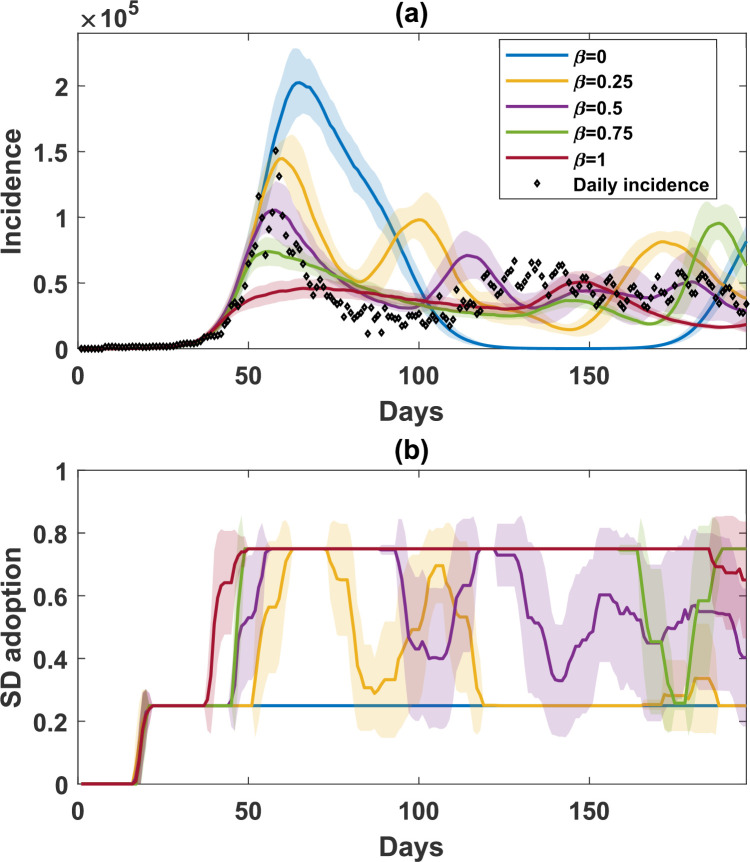
A comparison of simulated incidence (a) and SD-adoption fractions (b), with opinions produced by self-evaluation only varying β=[0,1] with 0.25 increment. Shaded areas around the solid line show standard deviation. Each simulated profile is averaged over 25 runs. The actual daily incidence is shown in scattered diamonds. We note that the purple profile (β=0.5) is equivalent to SE1 in figure 3.

Our results show that a moderate value of β=0.5 produced some recurrent waves (SE1: figure 3), but could not generate a satisfactory match to the observed magnitude of the first peak and the interval between waves. See §3.3 and figure 4 for a full investigation varying β between 0 and 1.

**Figure 5.**
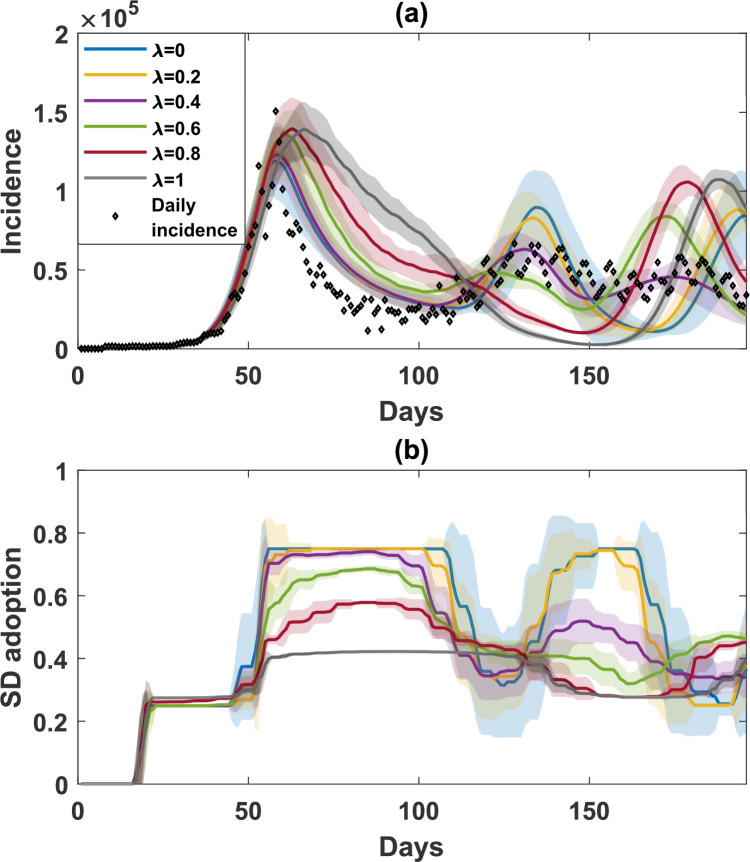
A comparison of simulated incidence (a) and SD-adoption fractions (b), with opinions produced by combining self-evaluation and peer pressure, varying the peer pressure weight λ=[0,1] with increment 0.2. The considered social contexts are HH and WG, with equal weights ($\psi^{\text{HH}}=\psi^{\text{WG}}=0.5$). Note that λ=0 is equivalent to SE3 in figure 3. Shaded areas around the solid line show standard deviation. Each simulated profile is averaged over 25 runs. The actual daily incidence is shown in scattered diamonds.

**Figure 6.**
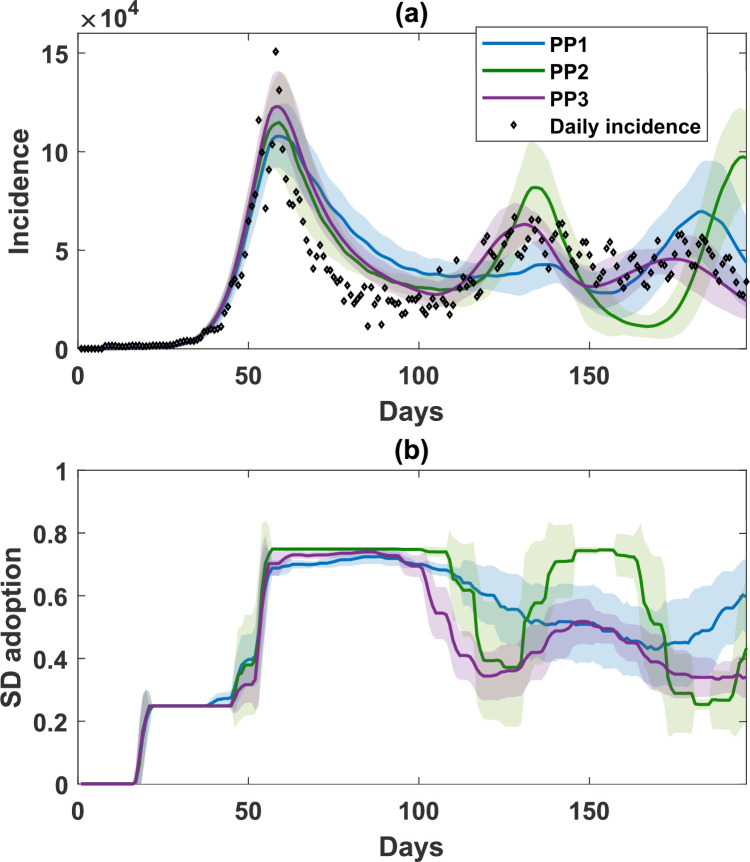
A comparison of (a) simulated incidence and (b) SD-adoption fraction, with opinions produced by combining self-evaluation and peer pressure in different social contexts (PP1, household only, in blue; PP2, working groups only, in green; and PP3, household and working groups with equal weight, in purple). Parameters relating to self-evaluation are set to the same values as in SE3 (see table 1). Shaded areas around the solid line show s.d. Each simulated profile is averaged over 25 runs. The actual daily incidence between December 2021 and June 2022 is shown in scattered diamonds.

By introducing the time-dependent memory horizon (SE2: figure 3), we observed improved accuracy in reproducing the second wave, but the difference with the first incidence peak could not be reconciled. We found that the combined effect of the time-dependent memory horizon and pandemic fatigue (SE3: figure 3) could reproduce the first peak and the timing of the second wave, but not the amplitude of the second incidence peak. These results suggested that the complex pandemic dynamics (e.g. recurrent waves with a prominent first wave and slower subsequent waves) observed during the Omicron stage in Australia could not be reproduced by our opinion dynamics model based solely on self-evaluation of risks.

### 3.2. Partial peer pressure

To examine the peer pressure component coupled with scenario SE3, we explored the impact of the peer pressure weight λ, while selecting different social contexts. Specifically, we modelled three scenarios where peer pressure originated from household (PP1, $\psi^{\text{HH}}=1$), working group (PP2, $\psi^{\text{WG}}=1$), and both household and working group with equal influence (PP3, $\psi^{\text{HH}}=\psi^{\text{WG}}=0.5$). Parameter settings in these scenarios are summarized in table 1. The investigation varying λ between 0 and 1 is described in §3.3.

**Table 1.** Simulation scenarios of opinion dynamics using self-evaluation and peer pressure. HH = household, WG = working groups.

self-evaluation			
simulation	β	γ	*T* (days)
SE1	0.5	0	7
SE2	0.5	0	following equation (2.3)
SE3	0.5	0.002	following equation (2.3)

peer pressure			
simulation	λ	^ $\psi^{\text{HH}}$ ^	^ $\psi^{\text{WG}}$ ^
PP1	0.4	1	0
PP2	0.4	0	1
PP3	0.4	0.5	0.5

We observed that adding the peer pressure weight λ affected the number of waves and the corresponding incidence peaks (figure 5), with a mid-range λ=0.4 providing the best match. The selection of different peer-pressure forming social contexts (HH, WG or both) did not affect the first wave, with all scenarios demonstrating a high SD adoption in response to the prominent first peak (figure 6). However, the choice of peer-pressure social contexts affected the second and third pandemic waves. Specifically, opinions inferred from a smaller social context (HH, with between 1 and 6 household members; PP1) led to a smaller variability in SD adoption after the first incidence peak, resulting in a slower decline in SD adoption and consequently, a lower second incidence peak.

On the other hand, opinions inferred from a larger social context (WG, up to 20 co-workers; PP2), accentuated fluctuations in the SD adoption, resulting in more drastic oscillatory incidence dynamics during the second and third waves. When the peer pressure originated from both contexts HH and WG (PP3), the resultant pandemic dynamics were closest to the observed incidence, showing three distinct waves: an acute, high incidence peak around day 60, followed by two smaller incidence peaks around day 130 and day 170 (see table 2 for the comparison between simulated and actual incidence dynamics).

**Table 2.** Mean incidence peaks and the corresponding simulation day in the considered simulated scenarios.

scenario	first peak (day)	second peak (day)	third peak (day)
actual	150 700 (58)	66 780 (128)	58 080 (177)
SE1	105 500 (58)	71 020 (114)	41 680 (151)
SE2	99 740 (58)	64 400 (145)	not formed
SE3	120 800 (59)	87 420 (135)	not formed
PP1	107 900 (59)	42 750 (138)	97 430 (195)
PP2	114 900 (59)	81 890 (134)	69 690 (183)
PP3	122 900 (58)	63 210 (131)	45 590 (174)

These findings were in concordance with our previous study [20]: the fluctuating adoption of SD strongly contributed to the recurrent waves observed during the Omicron pandemic stage in Australia. In this study, we further demonstrated that the changes in the SD adoption can themselves be attributed to several opinion-forming factors: risk aversion, memory horizon, perception fatigue, peer pressure, as well as selection of the peer-pressure forming social context(s).

### 3.3. Sensitivity analysis

We performed local sensitivity analysis to evaluate the response of an ‘output’ variable of interest, for example, incidence, to the change in an ‘input’ parameter. In prior work, we established robustness of the model through extensive global and local sensitivity analyses of key epidemiological parameters [20,31,32,34,36]. Here, we focus on opinion-related parameters and vary three parameters of interest: (i) β (risk aversion), (ii) λ (peer pressure weight), and (iii) proportion of the compliant and non-compliant agents. While the first two parameters affect the reactive opinion dynamics, the last parameter determines the fraction of the population whose opinion is independent of the severity of the pandemic.

We tested five different values of β. Figure 4 shows that β strongly influenced incidence waves and the associated peaks. While lower β (lower risk aversion) led to more frequent changes in both SD adoption and the incidence waves, higher β (higher risk aversion) resulted in a more stable SD adoption associated with a reduced number of waves at lower incidence peaks. We note that the extreme cases of β failed to produce multi-wave patterns. Specifically, β=0 produced a single-wave pandemic, with the majority of the population, 75%, not adopting SD (figure 4, blue profile), while β=1 produced endemic-like dynamics without acute peaks, with all rational agents choosing to adopt SD, i.e., 75% of SD adoption (figure 4, dark red profile).

We then varied the peer pressure weight λ between 0 and 1 at a 0.2 increment, as illustrated in figure 5. We found that higher λ encouraged the opinion convergence away from SD adoption, resulting in a prolonged first wave while delaying the second wave When λ=0 (figure 5, blue profile), peer pressure had no impact on the perception of the infection risk and the opinions solely depended on self-evaluation. Conversely, when λ=1 (figure 5, grey profile), the opinion formation was purely dependent on the average opinion of the selected opinion-affecting social contexts.

**Figure 7.**
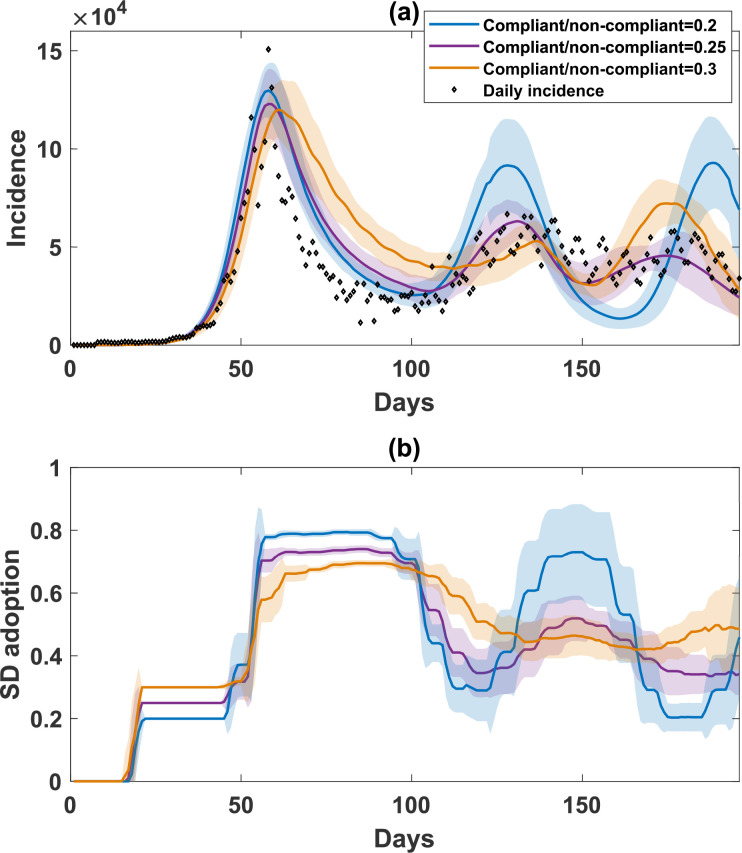
A comparison of simulated incidence (a) and SD-adoption fractions (b) with opinions produced by combining self-evaluation and peer pressure, varying the proportion of compliant and and the proportion of non-compliant agents from 0.2 to 0.3 with increment 0.05. Opinion-related parameters are constant across all three cases (β=0.5, λ=0.4, $\psi^{\text{HH}}=\psi^{\text{WG}}=0.5$). We note that the purple curve is equivalent to PP3 in figure 6. Shaded areas around the solid line show s.d. Each simulated profile is averaged over 25 runs. The actual daily incidence is shown with scattered diamonds.

**Figure 9.**
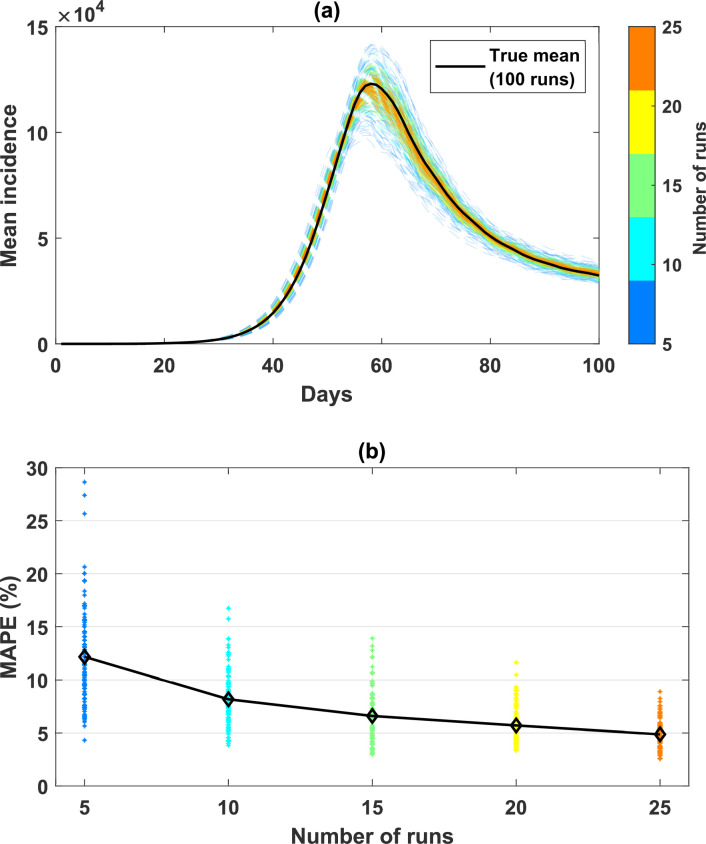
A comparison of simulated incidence generated across different numbers of runs for 100 simulation days. (a) Mean incidence. Each dashed profile represents the mean incidence generated over a randomly sampled set of runs (between 5 and 25 at an increment of 5; refer to colour bar). Hundred sets are sampled per configuration. The ‘true’ simulated incidence (solid black line) is the mean trajectory computed over 100 runs. (b) The relationship between the mean absolute percentage error (MAPE) and the number of runs. Input parameters are equivalent to SE3 in figure 3.

**Figure 10.**
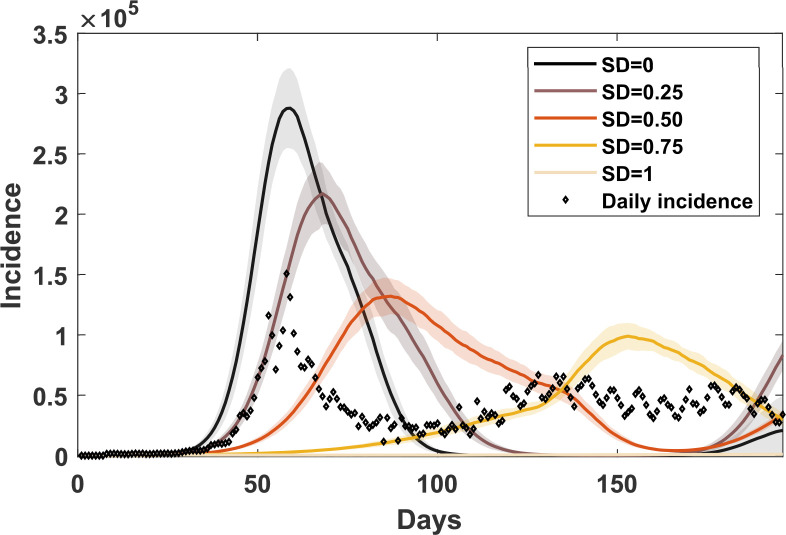
A comparison of simulated incidence in controlled and null cases, obtained by varying SD adoption between 0 and 1 at 0.25 increment. SD adoption of 0 represents the null case with zero adoption of SD, whereas SD adoption of 1 represents full adoption of SD by the population. Shaded areas around the solid line show standard deviation. Each simulated profile is averaged over 25 runs. The actual daily incidence between December 2021 and June 2022 is shown with scattered diamonds.

**Figure 11.**
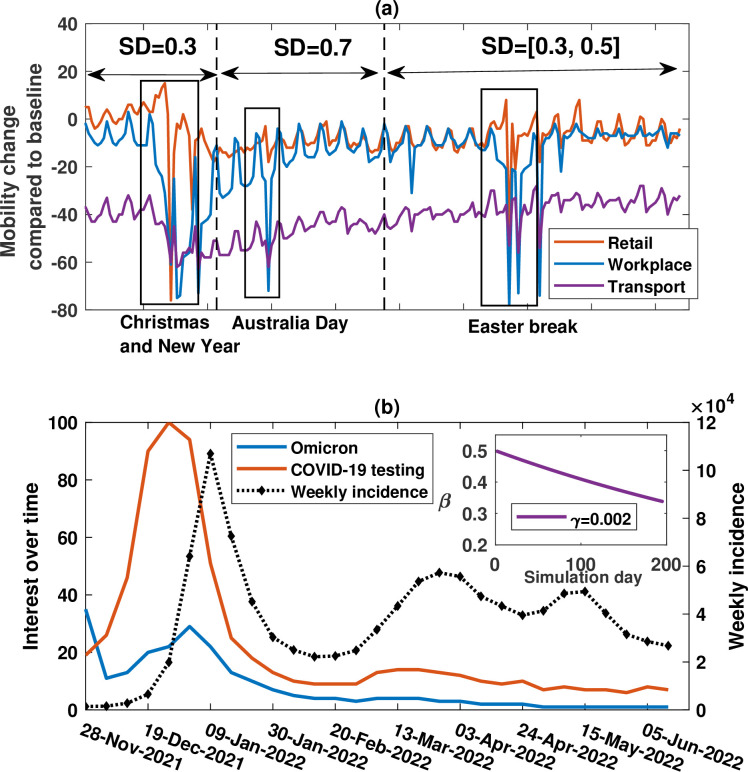
Movement trends and Google Trends during Omicron stage in Australia. (a) Movement trends compared to the baseline (the median value from the 5 week pre-pandemic period between 3 January and 6 February 2020). The vertical black lines mark the SD profile produced by opinion dynamic modelling shown in figure 6 (PP3). Public holidays are marked in black boxes. (b) Weekly Google Trends (*y*-axis, left) obtained for COVID-19-related keywords. Weekly incidence is shown as dotted black line (*y*-axis, right). Online interest over time represents the search interest in the given period where 100 indicates the peak popularity and lower values correspond to no popularity. The inset shows how the risk aversion parameter β declines over time given the fatigue rate γ=0.002.

Lastly, we varied the proportion of compliant and non-compliant agents, assuming that these cohorts take up an equal proportion of the population. We varied these proportions between 0.2 and 0.3 at a 0.05 increment; in other words, the remainder of the population (i.e. 0.6, 0.5 and 0.4, respectively) are specified as rational agents whose opinions change depending on the current pandemic severity and peer pressure. Figure 7 shows that the higher proportion of compliant and non-compliant agents (0.3) results in less abrupt changes in incidence waves and SD adoption due to the lower proportion of rational agents in the population. Nevertheless, all three simulated settings produce recurrent waves, showing that the model is robust to these variations.

## 4. Conclusions

Recurrent pandemic waves are a systemic phenomenon observed during the spread of many infectious diseases, including during the COVID-19 pandemic [44]. One of the factors contributing to this phenomenon is the fluctuating adoption of non-pharmaceutical interventions, such as SD [20]. To effectively capture changes in social behaviour, infectious disease models need to account for the rapid behavioural shifts driven by individual decision-making [12]. Following this approach also offers a quantitative insight into the nonlinear relationships and feedback loops between the pandemic spread and the compliance/adoption of SD behaviour driven by public opinion.

To address these questions, we modelled the dynamics of opinions which shape the decisions on voluntary SD adoption, using a large-scale pandemic ABM. Our integrated model captured complex feedback between opinion-driven SD adoption and pandemic spread, producing a fluctuating SD-adoption profile that generated recurrent pandemic waves. The identified SD-adoption profile is in qualitative agreement with the actual mobility reduction during this period that was reported across the workplace, retail, and transport [45]. Our prior study [20] retrospectively selected six SD-adoption fractions in a sequence of five-step changes, while minimizing the difference between simulated and actual incidence curves. In contrast, the opinion dynamic model proposed and validated in this study produced a more intuitive and constructive explanation of voluntary SD adoption, using five underlying factors, attributing the varying SD adoption to a combined effect of individual risk aversion and social peer pressure. We identified an opinion formation scenario that best matches the recurrent waves observed during the Omicron stage in Australia by fine-tuning these five factors: risk aversion β, fatigue γ, memory horizon T, peer pressure weight λ, and selected opinion-affecting social contexts $\psi_{g}$. Although flexible and voluntary SD increases personal freedom, they are also associated with recurrent waves of pandemic incidence, albeit with smaller peaks in subsequent cases. This situation highlights the challenging trade-offs between ethical public health interventions, individual rights and effective public health mitigation strategies.

In contrast with many pandemic opinion dynamics models, our model includes a heterogeneous population, with agents interacting both in terms of both infection transmission and opinion dynamics. This study extended our pandemic ABM [20,31–34] by incorporating re-infection and waning immunity.

There are several limitations of this work. First, we assumed that all agents use the normalized nationwide incidence (i.e., ‘global’ incidence) to form the perceived risk of infection with identical risk aversion parameter β and fatigue rate γ. This simplification did not consider scenarios where (i) agents may have different risk aversion levels and perception fatigue rates (i.e. β and γ distributed non-uniformly across the population), and (ii) agents may use ‘local’ incidence (e.g. from the local health district, city, or state) during the risk evaluation process. Future work can address these limitations by allowing β to follow a power law distribution accounting for extreme risk-averse or risk-seeking behaviours in the population. In addition, β can also be correlated with an agent’s infection status and immunity level, for example, allowing β to change pre- and post-infection and/or immunization.

Second, we interpreted peer pressure as the average opinion across specific social contexts where all opinions are weighted equally. In reality, however, some members of the community may have greater influence than others (e.g. opinion leaders and community champions). One possible future direction is to model several agents assigned a higher weight of opinion affecting a large social context (e.g. community) and explore the impact of these agents on the coupled pandemic and opinion dynamics. We also acknowledge that there are other methods to model peer pressure, for example, conformity [29].

Lastly, we established robustness of the model through local sensitivity analysis by varying one parameter at a time. Robustness of the model can be further studied by performing global sensitivity analysis through a multi-directional parameter space [46,47].

We also acknowledge that many intrinsic and extrinsic factors may contribute to the multi-wave phenomenon, including waning immunity, variant evolution and improving antigen testing policies. Our previous study [20] identified the changing levels of SD adoption as one of the factors capable of producing recurrent incidence waves. In this study, we investigated the feedback between opinion dynamics and behavioural changes in SD adoption, focussing specifically on varying the opinion dynamics factors. Although this study does not rule out biological factors that could impact the underlying transmission dynamics, it points to the importance of considering social dynamics and feedback as a key driver of epidemic outcomes.

Overall, we quantitatively modelled a nonlinear interplay between opinion dynamics and pandemic spread in a heterogeneous population, revealing a balanced impact of individual risk perception and social peer pressure on voluntary SD-adoption decisions and resultant recurrent pandemic waves. In contrast to previous studies [20,31], our model incorporates individual risk assessment within the context of public opinion regarding SD adoption. We believe that gaining a deeper understanding of the complex factors influencing opinion dynamics during pandemics can aid ethical decision-making and help policymakers develop effective, ethically aware crisis communication and response approaches.

## Figures and Tables

**Figure 8.**
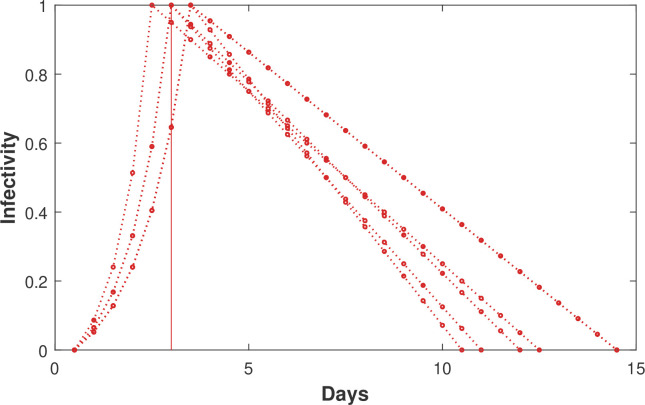
Model of natural disease history of the COVID-19 for the Omicron variant. The illustrated profiles are sampled from five random agents. Each profile rises exponentially until reaching the infectivity peak, followed by a linear decrease until full recovery. Vertical lines mark the mean incubation period, with the mean following a log normal distribution. The mean incubation period, recovery period and $R_{0}$ are summarized in table 3.

## Data Availability

AMTraC-19 v9.0 is open-source software and can be accessed on Zenodo: [48].

## Appendix A. AMTraC-19 modelling framework

### A.1. Population generation

We generated an artificial population representing the main demographic characteristics and commuting network of the Australian populatiA.1. oA.1. n, by using several high-resolution datasets, including the Australian Census of 2021 [49], international air traffic reports with incoming passenger flows in Australian airports [50] and educational registration records including the number of schools and students [51]. The population generation process assigned each agent with multiple demographic characteristics (e.g. age, gender, residency location) and social contexts in different settings: residential (e.g. household, household cluster, etc.); educational (e.g. classroom/school for agents aged 18 years or younger); and workplace (for agents aged over 18 years). While the residential contexts are determined by residential demographics, the workplace and educational contexts follow the commuting network (travel to work and travel to school respectively, depending on agents’ age groups) [52]. The detailed up-to-date description of the population generation process can be found in supplementary materials of a recent study [34] and the user guide of our open-source software [48].

### A.2. Disease transmission

Using the generated surrogate population, we stochastically simulate infection transmission based on interactions within the agents’ social contexts. If an agent in susceptible state is exposed to the infection within one or more of its social contexts, it may get infected (dependent on infection probabilities), and then progress through several states: latent, infectious (asymptomatic or symptomatic) and recovered. We assumed no population dynamics during the simulated period (i.e. no births and deaths).

We use the daily probability of interaction $q_{j\to i}(g)$, potentially transmitting infection from agent j to agent i, which depends on the ages of agents j and i, and on the context g in which the interaction occurs. These probabilities $q_{j\to i}(g)$ have been defined and calibrated in previous studies [20,31].

At cycle n, given the interaction probabilities $q_{j\to i}(g)$, we define the transmission probability $p_{j\to i}(n,g)$ which depends on the epidemiological characteristics and natural history of the disease:

(A 1)
$$\begin{array}{r} p_{j\to i}(n,g)=\kappa \;f_{j}(n-n_{j})\;q_{j\to i}(g), \end{array}$$

where κ is a global transmission scalar used to calibrate the reproductive number $R_{0}$, $n_{j}$ denotes the infection onset time for agent j, and the agent-specific function $f_{j}(n-n_{j})$ is the natural history of the disease, reflecting the infectivity of agent j as its infection progresses as illustrated in figure 8. At the time cycle n, if agent j is not infected, $f_{j}(n-n_{j})=0$. If agent j is infected, $f_{j}(n-n_{j})\geq 0$.

**Table 3.** Model parameterization for Omicron variant adopted by AMTraC-19. The last two rows show the corresponding basic reproductive number and generation interval computed from the simulation, calibrated previously [20,34].

parameter	value/mean	distribution	description	range and reference
κ	23	constant	global transmission scalar	[21,25], ref. [20]
*T* ^rec^	3	log-normal(μ = 1.013, σ = 0.413)	incubation period (days)	
*T* ^rec^	9	uniform on [7,11]	recovery period (days)	ref. [31]
*T* ^lat^	0	constant	latent period (days)	
$\sigma^{\text{a}}$	0.67	constant	probability of symptoms, adults (age>18)	[0.47,0.87], ref. [20,31]
$\sigma^{\text{c}}$	0.268	constant	probability of symptoms, children (age≤18)	[0.067,0.268], ref. [32,36]
$\alpha_{asymp}$	0.3	constant	asymptomatic infectivity	[0.2,0.5], ref. [31,36]
π	0.12	constant	daily case detection probability, symptomatic	[0.1,0.12], ref. [20]
$\pi^{\prime }$	0.01	constant	daily case detection probability, asymptomatic	
*R*_0_	19.56	empirical	basic reproductive number	95% CI [19.12, 19.65], ref. [20,34]
*T*_gen_	5.42	empirical	generation interval (days)	95% CI [5.38, 5.44], ref. [20,34]

When a susceptible unvaccinated agent i, not protected by any NPIs, is exposed to the infection for the first time, the infection probability (i.e. probability of changing the agent state to latent) across all social contexts is calculated as:

(A 2)
$$\begin{array}{rl} p_{i}(n) & =1-\prod_{g\in G_{i}(n)}\;\prod_{j\in A_{g}\setminus \{i\}}\left(1-p_{j\to i}(n,g)\right), \end{array}$$

where $G_{i}(n)$ denotes the set of all social contexts g that agent i interacts with during the time cycle n, $A_{g}\backslash \{i\}$ denotes the set of agents in g (excluding agent i).

An infected agent can be either symptomatic or asymptomatic. The probability of symptomatic illness $z_{i}(n)$ is determined with respect to the infection probability $p_{i}(n)$, by incorporating an age-dependent scaling factor $\sigma_{i}$ that represents the fraction of symptomatic cases among the total cases:

(A 3)
$$\begin{array}{r} z_{i}(n)=\sigma_{i}\;p_{i}(n), \end{array}$$

where for adults (age>18) $\sigma_{i}=\sigma^{\text{a}}$; and for children (age≤18) $\sigma_{i}=\sigma^{\text{c}}$.

We calibrated the associated parameters (summarized in table 3) to match the characteristics of the Omicron variant following [20,34]. In this study, we also introduced re-infections, so that agents may become susceptible again upon recovery. The transition between recovered and susceptible states takes place after a fixed post-recovery period (e.g. 60 days), which aligns with clinical observation of re-infection periods for Omicron infections [53,54].

### A.3. Non-pharmaceutical interventions

NPIs may reduce the infection probability for susceptible agents. We characterize each NPI by (i) macro-distancing level, defined as the NPI-adopting fraction of all population, and (ii) micro-distancing level, defined as the adjusted interaction strengths between the NPI-adopting agents and other agents in the same social group (see table 4 for NPI parameterization).

**Table 4.** The macro-distancing parameters (population fractions) and micro-distancing (interaction strengths, *F(g)*) for the considered NPIs. The micro-distancing duration of CI is limited by the disease progression in the affected agent i, $D_{i}$. CI = case isolation, HQ = home quarantine, SC = school closures, SD = social distancing

	macro-distancing			micro-distancing			
intervention	adoption level	duration (days)	threshold (cases)	household	community	workplace or school	duration (days)
CI	0.7	196	0	1.0	0.25	0.25	$D_{i}$
HQ	0.5	196	0	2.0	0.25	0.25	7
SC students/teachers	1	110	100	1.0	0.5	0	110
SC parents	0.25	110	100	1.0	0.5	0	110
SD	dynamic	196	400	1.0	0.1	0.25	dynamic

At each time point, relevant NPIs incorporated in our model (CI, HQ, SD and SC) affect the agents according to their state, over a certain duration $D_{i}$. While CI and HQ are activated at the start of the simulation and last throughout the simulated period, SD and SC are triggered only if the national cumulative incidence exceeds a certain threshold. Assignment of NPI-adopting agents follows a Bernoulli process and an agent may adopt multiple NPIs. However, the interaction strengths are adjusted to the value of $F_{j}(g)$ following a descending priority assignment order: CI, HQ, SD and SC. In this study, we assumed a constant level of adoption with for CI, HQ and SC, while the SD adoption level is formed by the opinion dynamics detailed in §2. Therefore, there are no macro-adoption or micro-duration parameters for SD.

Formally, the probability of infection for NPI-adopting agents is extended from equation (A 2) as:

(A 4)
$$\begin{array}{rl} p_{i}(n)=1-\prod_{g\in G_{i}(n)}[ & 1-F_{i}(g)(1-\prod_{j\in A_{g}\setminus \{i\}}(1-F_{j}(g)\;p_{j\to i}(n,g)))], \end{array}$$

where $F_{j}(g)$ is the strength of the interactions between agent j and other agents in the social context g. For NPI-adopting agents j, the interaction strength is $F_{j}(g)\neq 1$. For non-adopting agents j, the interaction strength remains unchanged, $F_{j}(g)=1$. Note that equation (A 4) reduces to equation (A 2) if $F_{j}(g)=1$. The micro-distancing parameters for a context g are summarized in table 4.

### A.4. Vaccination

Vaccination reduces the infection risk for susceptible agents. In this study, we modelled a high pre-emptive vaccination coverage (90% of the population, or 22.89 million agents), attained before the Omicron stage. The vaccine distribution follows an age-dependent proportion [33]: 8.33% of vaccinated agents are aged 18 years or younger (age ≤18), 83.34% are aged between 18 and 65 years (18< age <65), and 8.33% are aged 65 years or older (age ≥65). Two types of vaccines are rolled out in our model, each covering 11.443 million agents (or 45% of the simulated population): the ‘priority’ vaccine with a higher clinical efficacy ($\eta^{\text{pri}}$), and the ‘general’ vaccine with a medium efficacy ($\eta^{\text{gen}}$). Table 5 shows the efficacy values aligned with clinical observations following the booster vaccination against the Omicron variant [55,56].

**Table 5.** Simulation parameters for reinfection and vaccine waning.

parameter	value	description
$\eta^{\text{pri}}$	0.7	clinical efficacy, priority vaccine
$\eta^{\text{gen}}$	0.5	clinical efficacy, general vaccine
$m^{\text{c}}$	0.7 or 0.5	peak immunity against symptomatic infection
$m^{\iota }$	0.4	peak immunity against infection transmission
ϵ	0.00034	immunity waning rate per cycle
$T^{\text{post}}$	60	post-recovery period before reinfection (days)

Following [20,32,33], we decomposed η into the susceptibility-reducing efficacy θ and the disease-preventing efficacy ζ as: η=θ+ζ−θζ.

### A.5. Waning immunity

The agents with vaccination and/or past infections can develop immunity against the disease, reducing their risk of infection [57]. In our model, for a susceptible agent i with a history of records $H_{i}$ containing all vaccinations and past infections r ($r\in H_{i}$), we assumed that the immunity from either vaccination or infection can be characterized by two separate immunity components: (i) compound immunity against symptomatic infection ($m_{i}^{\text{c}}$), and (ii) immunity against forward infection ($m_{i}^{\iota }$). At simulation cycle n, for every past record r, each immunity component wanes over time as follows:

(A 5)
$$\begin{array}{rl} m_{i}^{\text{c}}(n,r)=m^{\text{c}}(r) & [1-\text{min}(1,(n-n_{r})\epsilon )],\\ m_{i}^{\iota }(n,r)=m^{\iota }(r) & [1-\text{min}(1,(n-n_{r})\epsilon )], \end{array}$$

where ϵ is the immunity waning rate per simulation cycle, $n_{r}$ is the time cycle of past vaccination or infection record r, and $m^{\text{c}}(r)$ is the peak compound immunity developed from either vaccination ($m^{\text{c}}=\eta^{\text{pri}}$ or $m^{c}=\eta^{\text{gen}}$), or past infection ($m^{\text{c}}=0.7$) [58,59], while $m^{\iota }(r)$ is the peak immunity against forward transmission (see table 5). For agents with multiple vaccination and/or infection records, the compound immunity against symptomatic infection, denoted $M_{i}^{\text{c}}(n,H_{i})$, and the immunity against infection transmission, denoted $M_{i}^{\iota }(n,H_{i})$, are accumulated nonlinearly with an upper bound of perfect immunity of 1:

(A 6)
$$\begin{array}{r} M_{i}^{\text{c}}(n,H_{i})=\text{min}\left(\sqrt{\sum_{r\in H_{i}}{\left[m_{i}^{\text{c}}(n,r)\right]}^{2}},1\right)\\ M_{i}^{\iota }(n,H_{i})=\text{min}\left(\sqrt{\sum_{r\in H_{i}}{\left[m_{i}^{\iota }(n,r)\right]}^{2}},1\right). \end{array}$$

Similar to the vaccine efficacy, we decomposed compound immunity $M_{i}^{\text{c}}(n,H_{i})$ into susceptibility-reducing immunity ($M_{i}^{\theta }$) and disease-preventing immunity ($M_{i}^{\zeta }$):

(A 7)
$$\begin{array}{r} M_{i}^{\text{c}}(n,H_{i})=M_{i}^{\zeta }+M_{i}^{\theta }-M_{i}^{\zeta }M_{i}^{\theta }. \end{array}$$

Assuming that $M_{i}^{\zeta }=M_{i}^{\theta }$, we can derive $M_{i}^{\zeta }(n,H_{i})$ and $M_{i}^{\theta }(n,H_{i})$ as follows:

(A 8)
$$\begin{array}{r} M_{i}^{\zeta }(n,H_{i})=M_{i}^{\theta }(n,H_{i})=1-\sqrt{1-M_{i}^{c}(n,H_{i})} \end{array}.$$

The infection probability for a susceptible agent i, given their adoption with NPIs, the immunity levels, and the exposure to infection in all contexts $g\in G_{i}$, is then defined as follows:

(A 9)
$$p_{i}(n)=\left(1-M_{i}^{\theta }(n,H_{i})\right)[1-\prod_{g\in G_{i}(n)}[1-F_{i}(g)(1-\prod_{j\in A_{g}\setminus \{i\}}(1-(1-M_{j}^{\iota }(n,H_{j}))F_{j}(g)\;p_{j\to i}(n,g)))]].$$

Considering equation (A 9), we note that the infection probability $p_{i}$ is affected by both susceptibility-reducing immunity $M_{i}^{\theta }$ of the agent i and transmission-limiting immunity $M_{j}^{\iota }$ of the agent’s contacts j. The outer term $\left(1-M_{i}^{\theta }(n,H_{i})\right)$ represents the reduction in infection probability across possible contacts, given the agent i’s own susceptibility-reducing immunity $M_{i}^{\theta }$. The inner term $\left(1-M_{j}^{\iota }(n,H_{j})\right)$ potentially reduces the probability of infection, given a limited forward transmission from each agent j that can interact with i within their shared social context g.

The agent probability to become ill (symptomatic) is influenced by the disease-preventing immunity component, ($M_{i}^{\zeta }$):

(A 10)
$$\begin{array}{r} z_{i}(n)=\left(1-M_{i}^{\zeta }(n,H_{i})\right)\;\sigma_{i}\;p_{i}(n), \end{array}$$

where $\sigma_{i}$ is the fraction of symptomatic cases among total infection cases (see appendix A.6 for more details). Note that equation (A 9) reduces to equation (A 4), and equation (A 10), reduces to equation (A 3), if agent i has no prior immunity. Table 5 summarizes the parameters used in modelling waning immunity.

### A.6. Disease detection

We assumed that only a fraction of total infections are detected on a daily basis, given the voluntary self-reporting system adopted in Australia during 2021–2022. We also assumed that the asymptomatic cases are more difficult to detect than symptomatic cases so that detection probabilities π (symptomatic) and $\pi^{\prime }$ (asymptomatic) follow $\pi \gg \pi^{\prime }$. Table 3 includes the values of π and $\pi^{\prime }$ used in this study. These values are obtained following the approximation detailed below.

During the infection period, the probability that an infected agent i is detected within d days since the infection onset is defined as follows:

(A 11)
$$\begin{array}{r} \delta_{i}(d)=1-(1-\pi_{i}{)}^{d}, \end{array}$$

where $\pi_{i}$ is the probability of detection for agent i depending on their symptomaticity:

(A 12)
$$\begin{array}{r} \pi_{i}=\left\{ \begin{array}{ll} \pi & \text{if agent }i\text{ is symptomatic}\\ \pi^{\prime } & \text{if agent }i\text{ is asymptomatic} \end{array}\right.. \end{array}$$

The expected detection probability of an infected agent within d days since infection onset, accounting for all possible infections (asymptomatic or symptomatic), is then given by:

(A 13)
$$\begin{array}{rl} E[\delta_{i}(d)] & =\sigma_{i}\left(1-(1-\pi {)}^{d}\right)+(1-\sigma_{i})\left(1-(1-\pi^{\prime }{)}^{d}\right)\\ & =1-\sigma_{i}(1-\pi {)}^{d}-(1-\sigma_{i})(1-\pi^{\prime }{)}^{d}, \end{array}$$

where $\sigma_{i}$ is the probability that an infection in agent i is symptomatic (see equation (A 3)). We assumed that an infection is either symptomatic or asymptomatic from the onset.

We calibrated π and $\pi^{\prime }$ as follows. According to [37], the anti-nucleocapsid antibody prevalence elicited by natural infection was 46% (i.e. 2300) out of 5000 blood donation samples collected between 9 and 18 June 2022. Arguably, this approach underestimates the cases due to the systematic difference between samples from blood donors and those from the general population. To reduce the bias, we assume that (i) blood donation samples were only collected from susceptible but not infected individuals (including those recovered from previous infection) or asymptomatic individuals and, (ii) a constant ratio between symptomatic and asymptomatic cases, $\rho =\sigma^{\text{a}}/(1-\sigma^{\text{a}})$. We use the anti-nucleocapsid antibodies prevalence to estimate the recovered population nationwide:

(A 14)
$$\begin{array}{r} \frac{2300+\rho \times 2300}{5000+\rho \times 2300}\times 25.4\;\text{million}=18.256\;\text{million}, \end{array}$$

where the term ρ×2300 quantifies the corresponding number of symptomatic individuals present in the population at the time and not contributing to the blood donation.

Given the reported cumulative incidence of 7.3 million during this period [19], the fraction of detected infection cases nationwide can be approximated as: 7.3 million/18.256 million ≈ 40%. We found that a combination of π=0.12 and $\pi^{\prime }=0.01$ produced a satisfactory match at 37.8% after six days of detection, following equation (A 13) (assuming all testing occurs within six days of infection).

### A.7. Additional results

#### A.7.1. Simulation accuracy

To evaluate the accuracy of simulated dynamics over 25 runs, we computed the mean absolute percentage error (MAPE) [60] between the *‘true’* simulated incidence and the *‘sampled’* simulated incidence. To do so, we first established the true simulated incidence dynamics by computing the mean incidence over 100 runs (figure 9). Then, we randomly sampled a number of runs (between 5 and 25 at an increment of 5) and computed MAPE between the mean sampled simulated incidence and the true simulated incidence (figure 9b). Our results showed that MAPE reduced from 12.16% to 4.86% as the number of runs increased from 5 to 25. At 25 runs, the mean incidence is within 5% MAPE of the true incidence simulated over 100 runs, demonstrating an adequate level of accuracy.

#### A.7.2. Controlled and null cases

Appendix figure 10 illustrates that the controlled cases with SD adoption level at [0.25, 0.5, 0.75, 1] and the null case (SD adoption level equals 0), where SD adoption of 0 indicates no agents adopting SD and SD adoption of 1 indicates all agents adopting SD.

### A.8. Google mobility and online search trends data

Our simulated opinion dynamics and SD adoption fractions are in agreement with empirical evidence provided by the mobility [45] and online search trends [40], reported between 26 November 2021 and 10 June 2022. Using Google COVID-19 mobility reports for workplace, retail, and transport, we note a greater mobility reduction during the period when the model generated a higher SD adoption, indicating a reduced level of agent interactions (table 6 and figure 11a). In addition, we note that public interest in COVID-19-related topics declined during this time frame, as evidenced by the reduced popularity of keywords ‘Omicron’ and ‘COVID-19 testing’ reported in Google Trends (figure 11b). This observation matched our simulating scenario where the risk aversion of infection declined over time (i.e., declining β), governed by fatigue rate γ (figure 11b, inset).

**Table 6.** Mean mobility reduction. See figure 11 for the time series of mobility reduction.

category	SD = 0.3 26 Nov 2021–9 Jan 2022	SD = 0.7 10 Jan 2022–5 Mar 2022	SD = [0.3, 0.5] 6 Mar 2022–10 Jun 2022
workplace (%)	−9.6	−15.1	−9.9
transport (%)	−40.5	−46.5	−36.9
retail (%)	1.2	−9.6	−6.4

Notes
^1^
This includes the contrarian population estimated to be around 5% following the finding that ‘More than 1 in 20 believes that there is no hard evidence that COVID-19 actually exists’ [38], and essential workers estimated to comprise around 20% of the entire population, given that out of 14 million of the employed population, 40% work in essential industries and occupations [39]. That is, 40% × 14 million ÷ 25.4 million ≈ 20%.
